# Cardiac malignant peripheral nerve sheath tumor on computed tomography and magnetic resonance imaging: A case report

**DOI:** 10.1097/MD.0000000000017463

**Published:** 2019-11-01

**Authors:** Shuang Li, Yue Qiu, Jianqun Yu, Chunxiao Liang, Liqing Peng

**Affiliations:** a Department of Radiology, West China Hospital; b Department of Applied Mechanics, Sichuan University, Chengdu, China.

**Keywords:** computed tomography, heart neoplasms, magnetic resonance imaging, malignant peripheral nerve sheath tumor (MNPST)

## Abstract

**Rationale:**

Malignant peripheral nerve sheath tumor (MPNST) is a very rare sarcoma of the heart, and few cases have been reported. Herein, we retrospectively reviewed clinical manifestations, imaging features and management of our patient and other reported cases.

**Patient concerns:**

A 32-year-old woman was referred to the emergency department of our institution with expiratory dyspnea, edema of face for a month.

**Diagnosis:**

The patient was initially diagnosed with asthma at a local hospital based on a history of fatigue, cough and expiratory dyspnea, as well as negative electroencephalogram (ECG) and chest radiography. Based on computed tomography (CT) and cardiac magnetic resonance imaging (CMRI) in our hospital, she was found to have a malignant tumor involving right atrium. The tumor was diagnosed as MPNST according to histopathological results.

**Interventions:**

The tumor was deemed unresectable during the surgery. Then, the patient was referred for chemotherapy and radiotherapy.

**Outcomes:**

The patient deteriorated and died 4 months later.

**Lessons:**

Cardiac MPNST is very uncommon with nonspecific clinical and imaging characteristics according to limited cased reported. CMR, due to the high tissue resolution and multiple sequence imaging advantages, is useful for the detection, location and evaluation whether there is involvement of adjacent structures, and may help better clinical decision-making.

## 1 Introduction

Although malignant peripheral nerve sheath tumor (MPNST) ranks sixth of all soft-tissue sarcoma, accounting for approximately 5% to 10% of cases, MPNST of the heart is extremely rare with an incidence of 0.75% in all primary cardiac tumors.^[1,2]^ Due to the rarity, little is known about the features of cardiac MPNST. Cardiac computed tomography (CT) and magnetic resonance imaging (CMRI) are robust modalities in assessing cardiac morphology and function, and tissue characterization of myocardium. Herein, we report an MPNST located in the right atrium assessed by CT and CMRI. The imaging features of the case are presented and the utility of different imaging modalities in evaluation of this lesion is discussed.

## 2 Case report

A 38-year-old woman with a history of fatigue, cough and expiratory dyspnea for a month without obvious precipitating factor, who was referred to a local hospital. ECG and chest radiography were negative. She was diagnosed with asthma and treated with budesonide. The symptoms did not relieve but worsened with presentation of facial and bilateral limbs swelling. There were no other symptoms and no history of trauma, recent surgery and special disease, the related family history was absent too. Then she was referred to the emergency department of our institution. General physical examination was unremarkable. The laboratory tests showed marked elevation of CA-125 (184.5 u/ml), PRO-BNP (469 pg/ml) and lightly decrease of albumin (37.1 g/l).

**Figure 1:**
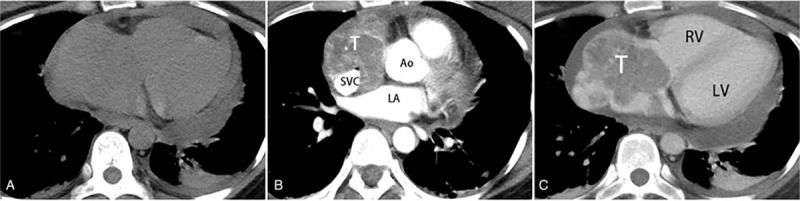
CT features of a malignant peripheral nerve sheath tumor in right atrium. (A) A lobulate mass (average CT value: 44 HU) locates in the RV with bilateral mild plural effusion and moderate pericardial effusion on CT plain scan. (B) At arterial phase, the mass is heterogenous and markedly enhanced (average CT value: 104 HU) with SVC being involved. (C) At the venous phase, the lesion tends to wash-out (average CT value: 78 HU). Note: T = tumor, SVC = superior vena cava, Ao = aorta, LA = left atrium, LV = left ventricle, RV = right ventricle, CT = computed tomography.

**Figure 2:**
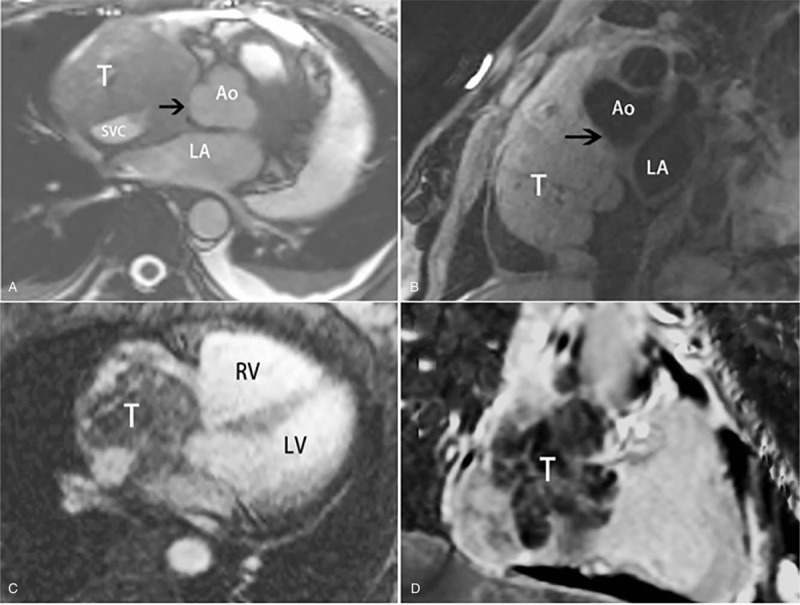
MRI features of the malignant peripheral nerve sheath tumor in right atrium. (A) Bright blood sequence, (B) T2-weighted images show an isointense and slight hyperintense mass attached to atrial right wall with a broad base, respectively. The fat plane between the mass and aorta is not clear and SVC is compressed. (C) First-pass perfusion demonstrates an ill-defined mass with fast perfusion. (D) The mass shows heterogeneous delayed contrast enhancement after gadolinium administration. Note: abbreviations as in Figure 1.

Transthoracic echocardiography (TTE) showed a hypoechoic mass in right atrium (71 × 38 mm) with moderate to severe pericardial effusion. CT revealed a large isodense right atrial mass with significant and inhomogeneous enhancement and showed superior vena cava and right auricle were involvement (Fig. 1). CMRI was further performed to seek to characterize the lesion. CMRI demonstrated a lobulated mass located in the right atrium and attached to atrial wall with a broad base. The mass was isointense on bright blood sequence and slight hyperintense on T2-weighted images, respectively (Fig. 2). After management of gadolinium, the lesion was ill-defined with fast perfusion on first-pass perfusion images and markedly heterogeneous contrast enhanced on late gadolinium enhancement images. Based on the imaging features mentioned above, it was diagnosed as a malignant tumor. It is difficult to make a definite diagnosis with nonspecific clinical manifestations and imaging features.

Surgery was scheduled for tumor resection. Median sternotomy was performed under general anesthesia. However, the tumor was found with invasion of the aortic root which was deemed unresectable and biopsies were taken for histopathological analysis. Histopathological examination indicated a spindle-cell malignant tumor. The immune-histochemical examination showed cells which were positive for CR, SMA (partly), S100 (partly), NSE, CD57 (partly), TLE-1, MIB-1 (partly), and negative for Myogenin, PCK, EMA, WT-1, CK56 and Des. The histopathological results were consistent with MPNST. After multiple disciplinary team discussion, the patient was referred for chemotherapy and radiotherapy. However, the therapeutic effect was unsatisfying, she refused to continue further treatment and died 4 months later.

## 3 Discussion

MPNST is defined as nerve sheath tumors arising from a peripheral nerve, or malignant tumors converting from benign neurogenic tumors.^[1,3]^ About half of cases happen to occur along with neurofibromatosis type 1 (NF-1), which is a genetic mutation on the 17th chromosome.^[1]^ As a result of the varying nomenclature in the past and different diagnostic criteria, few cases of cardiac MPNST have been reported. We performed a PubMed search using the term “(cardiac OR pericardial OR heart OR ventricle OR atrium) AND (Malignant peripheral nerve sheath tumor).” The cases meeting the following criteria were included in the present study: the reports were written in English and full-text was available; restricted to humans; the tumor originated from the heart chambers, myocardium, pericardium or the root of the great vessels; the final diagnosis was confirmed by histopathology.

Seven patients were included in this study (Table 1).^[2–8]^ So far, most relevant studies have focused on the pathologic manifestations. In this study, we mainly reported the clinical and imaging features of a cardiac MPNST, and we also retrospectively reviewed the literature and summarized the clinical data of other 7 published cases with cardiac MPNST.

**Table 1:**
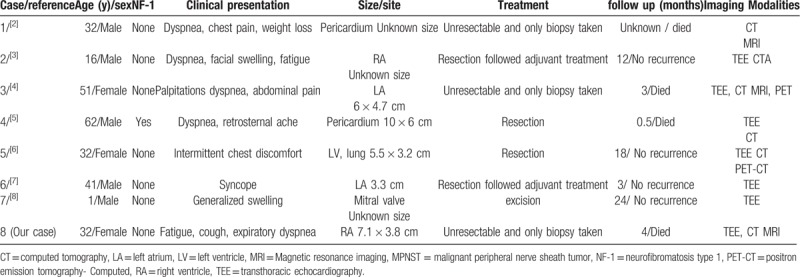
Literature review of cardiac MPNST and our case.

There were 5 males and 2 females in case reports. The mean age at diagnosed was 33.5 years (range from 24 months to 62 years) with five adults, a teenager (16 years) and an infant (23 months). The limited cases do not show any age and sex privilege for cardiac MNPST. The most common clinical manifestation is dyspnea (shortness of breath), with a frequency of 62.5% (5/8) including our case. Other clinical manifestations are quite nonspecific, including edema, chest pain, fatigue, weight loss and so on. The clinical presentations of cardiac tumors are nonspecific, mainly depending on the location and size of the mass.^[9]^ There were 4 lesions arising in the atrium (2 left, 2 right), and one in left ventricle, one in mitral valve and 2 in pericardium. Superior vena cava was obstructed in 3 cases, multi-chambers mass was seen in three cases and lung metastasis in one.

TTE, CT, and CMRI are common modalities used to evaluate cardiac masses. For the reported cases including our patient, 8 patients were elevated by TTE, 6 patients by CT, 3 by CMR and 2 patients with positron emission tomography-CT (PET-CT). As an initial imaging modality in evaluating a cardiac lesion, TTE can demonstrate the presence and location of a mass; however, the precise extension and nature of the lesion could not be clearly demonstrated due to the limited acoustic window sometimes. CT could depict more details of the mass. CMRI can locate the tumor precisely, and helpfully evaluate the extension and invasion of surrounding structures, and even characterize the lesion.

The CT imaging features of the published cases of MPNST were summarized in Table 2. All the masses in 6 patients with CT scans appeared as solid masses. Shapes of MPNST were variable, might be oval, irregular and lobulated. Sometimes, the mass could be tubular when it involves and extends into vena cava. Most cases (6/7, 85.7%) are ill-defined and showed infiltrative appearance indicating its malignant nature. In the reported series, tumor sizes were known in four cases ranging from 3.3 to 10 cm (mean, 6.2 cm). In our case, the size of the tumor is 7.1 cm, showing that MPNST is relatively large and could be easily detected by present imaging modalities. In our case, the lesion was an ill-defined lobulate soft tissue density mass with significant heterogenous enhancement. And for the reported cases, Cardiac MPNSTs were mostly seen as a slight lower density mass on unenhanced CT and significant or moderate heterogeneous enhancement after management of contrast agent. Our results suggest that Cardiac MPNST is more likely to present as an isodense or slightly hypodense on CT plain scan with moderate to significant enhancement.

**Table 2:**
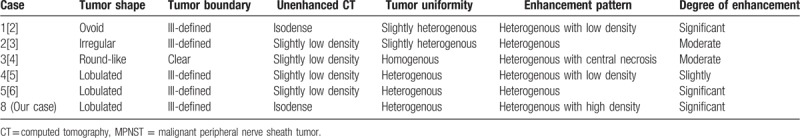
CT findings for 6 patients with cardiac MPNST.

Two patients reported with CMRI showed that the two masses were isointense on T1-weighted images and heterogenous on T2-weighted images. One of the 2 patients had contrast enhanced CMR study, and the mass was heterogeneous enhanced after gadolinium administration. In our case, the mass was isointense on bright blood sequence images and slightly hyperintensity on T2-weighted images with heterogenous and significant enhancement. However, the comprehensive CMR imaging features of cardiac MPNST may not be summarized due to the limited cases reported. Herein, we would like to emphasize the value of CMRI in evaluating the surgical resectability. With high tissue resolution, multiplanar and multiple sequence imaging features, CMRI can be very helpful to identify whether there is infiltration of adjacent tissue or structures. As for our case, the involvement of right auricle and superior vena cava was clearly demonstrated on CMRI, and there seemed no clear boundary between the mass and aorta. During surgery, the aortic root was proved to be affected.

Although making a definite diagnosis of MPNST can be difficult based on imaging alone, combined imaging examination is useful in distinguishing malignant tumor from a benign one, a distinction which is paramount for subsequent management. As Benz et al^[10]^ reported that the mean size of peripheral nerve sheath tumor was 4.8 ± 2.7 cm and MPNST was 7.4 ± 4.1 cm, showing that PNST is smaller than their malignant counterpart. Shurell et al^[11]^ used fludeoxyglucose F^18^ (FDG) PET imaging revealed that the mean maximum standardized uptake value (SUVmax) for MPNST was higher than that of benign one.^[11]^ MPNST, as an aggressive malignant tumor, can present invasive behaviors. For example, there were four patients showed moderate to severe pericardial or pleural effusion including our case. One of the patients, whose left lung was collapsed with severe left pleural effusion.

Complete excision of the tumors was considered the most effective treatment of MPNST. For cardiac sarcoma, even incomplete excision offers remission for patients.^[12]^ In the reported series of cardiac MPNST, 5 patients were referred to surgical treatment, 4 of whom were alive and doing well with 3 to 24 months of follow-up (mean, 14.5 months), 1 patient died 2 weeks after surgery. Two of them underwent followed adjuvant chemotherapy and local radiotherapy. But there were only small number of cases in our and previously published series, the effect of adjuvant chemotherapy and radiotherapy is difficult to judge. Three patients without surgery deteriorated and died 2 weeks to 4 months later. Cardiac MPNST is a common high-grade sarcoma with a relatively poor prognosis and has high risk of local recurrence and distant metastasis (40% to 65% of patients with local recurrence and 30% to 60% with metastasis).^[1]^

In summary, cardiac MPNST is very uncommon with nonspecific clinical and imaging characteristics according to limited cased reports. CMR, due to the high tissue resolution and multiple sequence imaging advantages, is useful for the detection, location and evaluation whether there is involvement of adjacent structures, and may help better clinical decision-making.

## 4 Ethical issues

The present study was approved by the Ethics Committee of the West China Hospital of Sichuan University. The patient information was anonymized and de-identified before analysis, who has provided informed consent for publication of the case.

## Author contributions

**Conceptualization:** Shuang Li, Yue Qiu, Liqing Peng.

**Investigation:** Shuang Li, Yue Qiu, Chunxiao Liang.

**Methodology:** Yue Qiu.

**Writing – original draft:** Shuang Li.

**Writing – review & editing:** Shuang Li, Yue Qiu, Jianqun Yu, Liqing Peng.
